# Comparison between vision transformers and convolutional neural networks to predict non-small lung cancer recurrence

**DOI:** 10.1038/s41598-023-48004-9

**Published:** 2023-11-23

**Authors:** Annarita Fanizzi, Federico Fadda, Maria Colomba Comes, Samantha Bove, Annamaria Catino, Erika Di Benedetto, Angelo Milella, Michele Montrone, Annalisa Nardone, Clara Soranno, Alessandro Rizzo, Deniz Can Guven, Domenico Galetta, Raffaella Massafra

**Affiliations:** 1 Struttura Semplice Dipartimentale Fisica Sanitaria, I.R.C.C.S. Istituto Tumori ‘Giovanni Paolo II’, Viale Orazio Flacco 65, 70124 Bari, Italy; 2 Unità Operativa Complessa di Oncologia Toracica, I.R.C.C.S. Istituto Tumori ‘Giovanni Paolo II’, Viale Orazio Flacco 65, 70124 Bari, Italy; 3 Unità Operativa Complessa di Oncologia Medica, I.R.C.C.S. Istituto Tumori ‘Giovanni Paolo II’, Viale Orazio Flacco 65, 70124 Bari, Italy; 4 Dipartimento di ElettronicaInformazione e Bioingegneria, Politecnico di Milano, Via Giuseppe Ponzio, 34, 20133 Milan, Italy; 5 Unità Operativa Complessa di Radioterapia, I.R.C.C.S. Istituto Tumori ‘Giovanni Paolo II’, Viale Orazio Flacco 65, 70124 Bari, Italy; 6 Unità Operativa Complessa di Oncologia Medica ‘Don Tonino Bello’, I.R.C.C.S. Istituto Tumori ‘Giovanni Paolo II’, Viale Orazio Flacco 65, 70124 Bari, Italy; 7 Department of Medical Oncology, Hacettepe University Cancer Institute, 06100 Sihhiye, Ankara, Turkey

**Keywords:** Lung cancer, Oncology, Computational science

## Abstract

Non-Small cell lung cancer (NSCLC) is one of the most dangerous cancers, with 85% of all new lung cancer diagnoses and a 30–55% of recurrence rate after surgery. Thus, an accurate prediction of recurrence risk in NSCLC patients during diagnosis could be essential to drive targeted therapies preventing either overtreatment or undertreatment of cancer patients. The radiomic analysis of CT images has already shown great potential in solving this task; specifically, Convolutional Neural Networks (CNNs) have already been proposed providing good performances. Recently, Vision Transformers (ViTs) have been introduced, reaching comparable and even better performances than traditional CNNs in image classification. The aim of the proposed paper was to compare the performances of different state-of-the-art deep learning algorithms to predict cancer recurrence in NSCLC patients. In this work, using a public database of 144 patients, we implemented a transfer learning approach, involving different Transformers architectures like pre-trained ViTs, pre-trained Pyramid Vision Transformers, and pre-trained Swin Transformers to predict the recurrence of NSCLC patients from CT images, comparing their performances with state-of-the-art CNNs. Although, the best performances in this study are reached via CNNs with AUC, Accuracy, Sensitivity, Specificity, and Precision equal to 0.91, 0.89, 0.85, 0.90, and 0.78, respectively, Transformer architectures reach comparable ones with AUC, Accuracy, Sensitivity, Specificity, and Precision equal to 0.90, 0.86, 0.81, 0.89, and 0.75, respectively. Based on our preliminary experimental results, it appears that Transformers architectures do not add improvements in terms of predictive performance to the addressed problem.

## Introduction

Non-small cell lung cancer (NSCLC) represents the most frequent form of lung cancer, treated mainly with surgery and modern radiotherapy^1–3^. Therapeutic approaches for NSCLC patients differ according tothe histological characteristics of the tumor and the patient's condition. The treatment path for patients with locally advanced NSCLC currently includes chemoradiotherapy possibly followed by immunotherapy. For early-stage patients, however, surgical resection followed by chemotherapy currently remains the only potentially curative treatment. Nonetheless, 30–55% of these patients develop post-resection tumor recurrence within the first 5 years^2^. Therefore, the early identification of patients most prone to developing a recurrence is a challenge that is currently still open and would allow clinicians to plan a more accurate therapeutic surveillance plan.

Several works have been proposed on the prediction of recurrence-free survival and overall survival in NSCLC patients. However, the state-of-the-art is lacking of models designed for the early prediction of disease recurrence. Furthermore, although all proposed models show encouraging results, they are still not suitable for a clinical application, even when they involve genomic-based models which are expensive and time-consuming procedures. In recent years, artificial intelligence has already demonstrated its potential in defining predictive and prognostic models. Specifically, the predictive power of radiomic features extracted from biomedical images is now well established in the scientific community^4–8^.

Recently, radiomics via Convolutional Neural Networks (CNNs) has been extensively used showing strong potential^5–20^. CNNs can be of two types: custom or pre-trained. In the former, scientists build their own network which is then trained to execute a specific task; in the latter case, a transfer-learning approach is used^15–20^. Networks are first trained on millions of images of different classes (e.g., ImageNet) in recognizing specific patterns like edges, dots, color gradients, shapes, etc.^21^. After that, this gained knowledge is transferred to the specific set of images to study. In this work, we adopted only the transfer learning approach. Typically, CNNs consist of several layers of convolutions and max pooling. When applied to images, the bottom layers (close to the input layer) focus on local simple features like edges, dots, and color gradients; higher layers, instead, combine the previous features into more complex ones and can be used to train Machine Learning models.

However, CNNs require high computational resources; second, they focus more on the entire image instead of its portions which could contain the lesion^22, 23^.

In 2020, the first ViT architecture was introduced and after that, a variety of different architectures appeared^24–40^. Differently from CNNs, ViTs consist of a small number of layers and can decompose the image in patches gaining information with the attention mechanism^37–40^. They turned out to reach promising performances even outperforming traditional CNNs^22, 23, 41–48^.

In this scenario, in light of innovative algorithms proposed in the literature, the aim of our work was to compare the performances of different state-of-the-art deep learning algorithms to predict disease recurrence in NSCLC patients. To the best of our knowledge, the state-of-the-art lacks a comparative study on the classification performances obtained by these two architectural families in relation to the problem of disease recurrence prediction evaluated on the same reference dataset. This information would allow us to lay the foundations for future studies aimed at defining and validating an accurate model of personalized medicine. Therefore, in this preliminary work, we used various Transformer architectures to predict NSCLC recurrence^14, 49–52^. We used a public database of CT images of 144 NCSLC patients for recurrence classification comparing the performances of ViTs and CNNs^53^. The paper is organized as follows: in Section “Results”, Materials and Methods, we introduce the database of patients and the network architectures; then, in Sections “Discussion and conclusion” and “Materials and methods”, Results and Discussion, we present the results of our transfer-learning-based model, discussing their performances.

## Results

**Figure 1:**
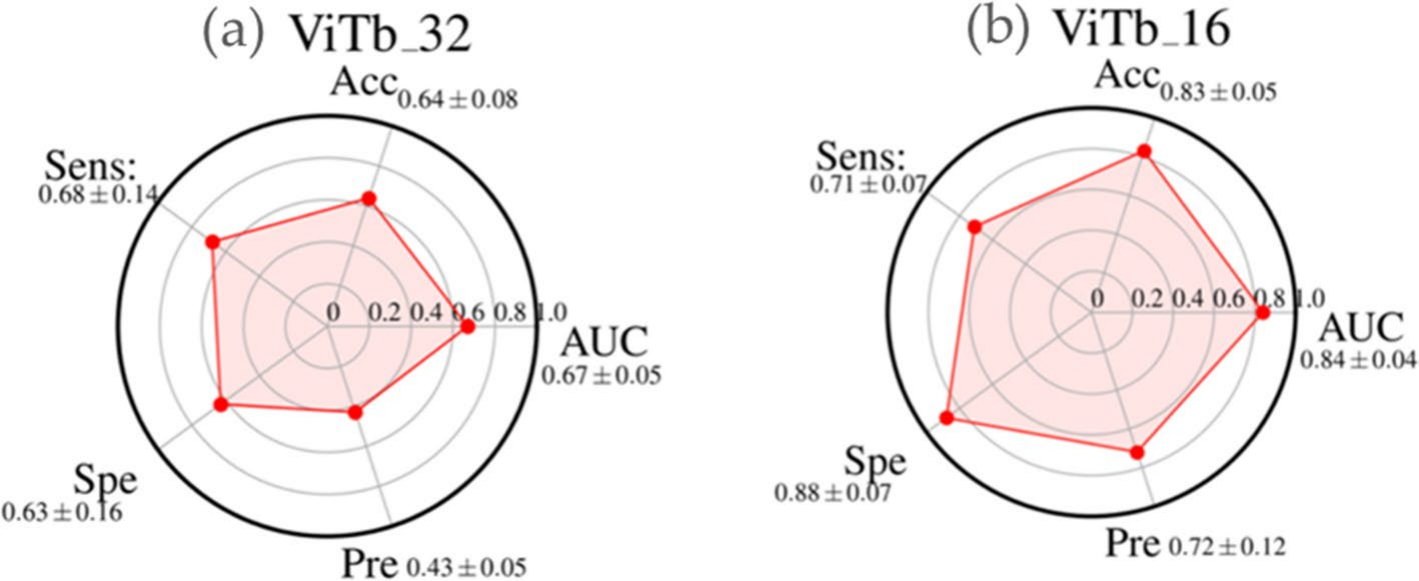
Radar plots of the performances AUC, Accuracy (Acc), Sensitivity (Sens), Specificity (Spe), and Precision (Pre) of ViTb_32 (**a**) and ViTb_16 (**b**). For each metric, the mean value, among all the cross-validation 20 rounds, is shown with its standard deviation.

**Figure 2:**
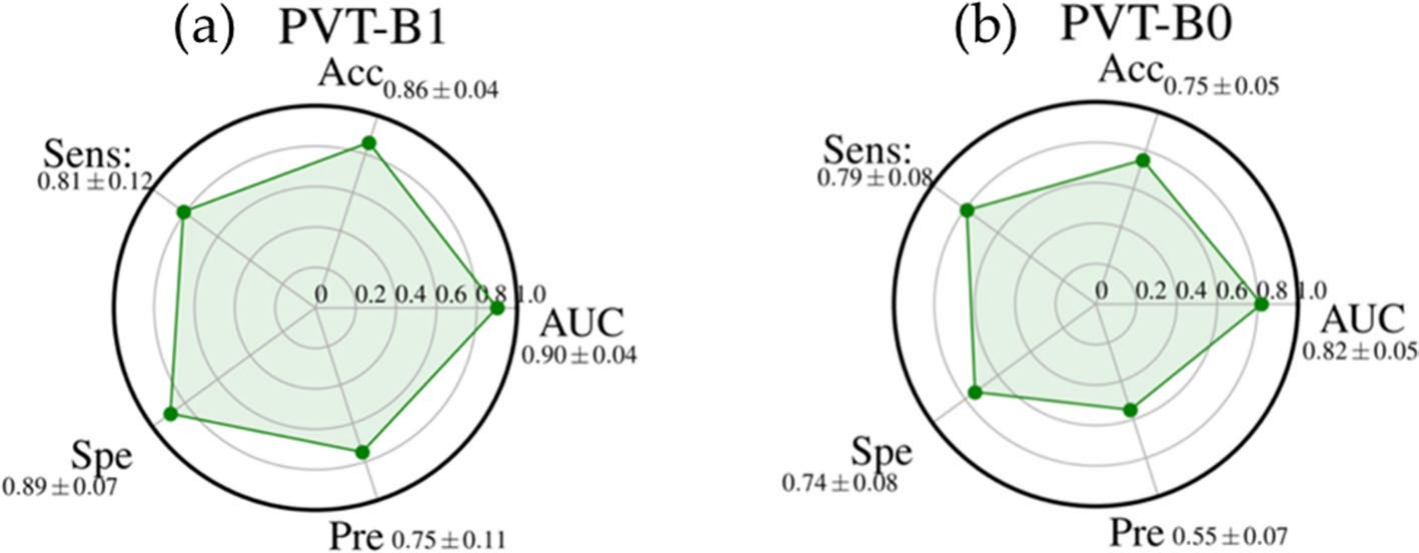
Radar plots of the performances AUC, Accuracy (Acc), Sensitivity (Sens), Specificity (Spe), and Precision (Pre) of PVT-B1 (**a**) and PVT-B0 (**b**). For each metric, the mean value, among all the cross-validation 20 rounds, is shown with its standard deviation.

**Figure 3:**
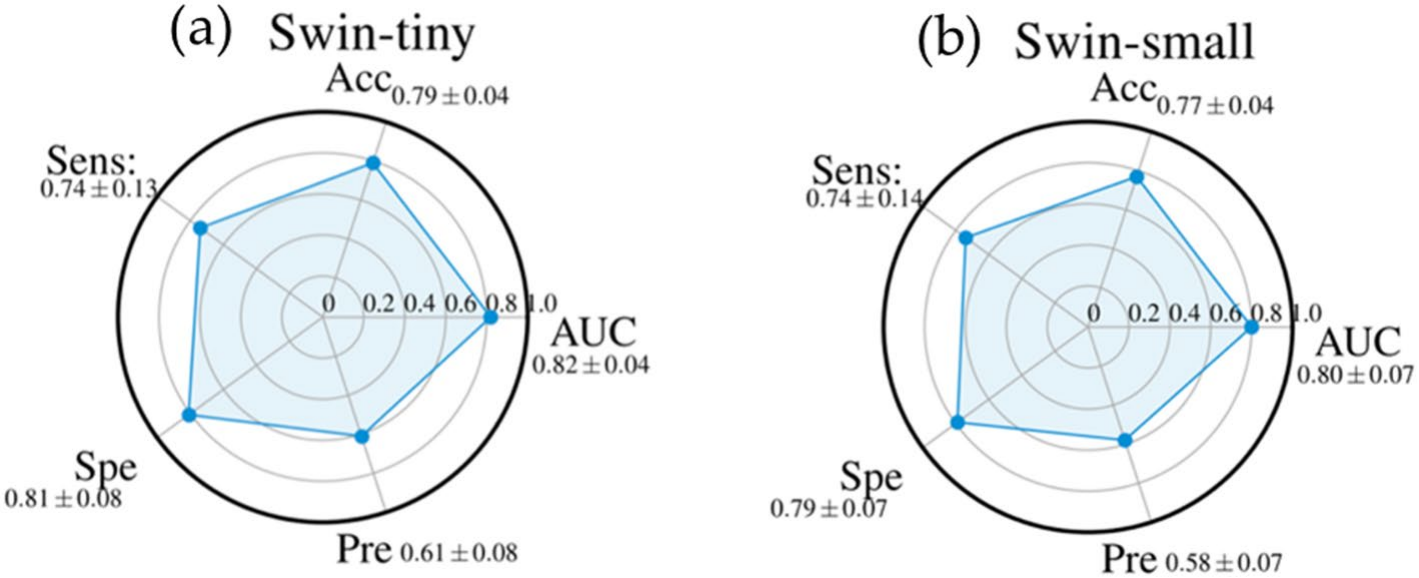
Radar plots of the performances AUC, Accuracy (Acc), Sensitivity (Sens), Specificity (Spe), and Precision (Pre) of Swin-tiny (**a**) and Swin-small (**b**). For each metric, the mean value, among all the cross-validation 20 rounds, is shown with its standard deviation.

The performances of diverse Transformer families are summarized in the radar plot of Figs. 1, 2, and 3: ViTb_32 and ViTb16 (Fig. 1a,b), PVT-B1 and PVT-B0 (Fig. 2a,b), Swin-tiny and Swin-small (Fig. 3a,b).

Among all the structures evaluated for this family of architectures, PTV_B1 shows the best performance (Fig. 2). It was highly performing with an AUC value, accuracy sensitivity, specificity and precision of 0.90 ± 0.04, 0.86 ± 0.04, 0.81 ± 0.12, 0.89 ± 0.07, and 0.75 ± 0.11 respectively.

**Figure 4:**
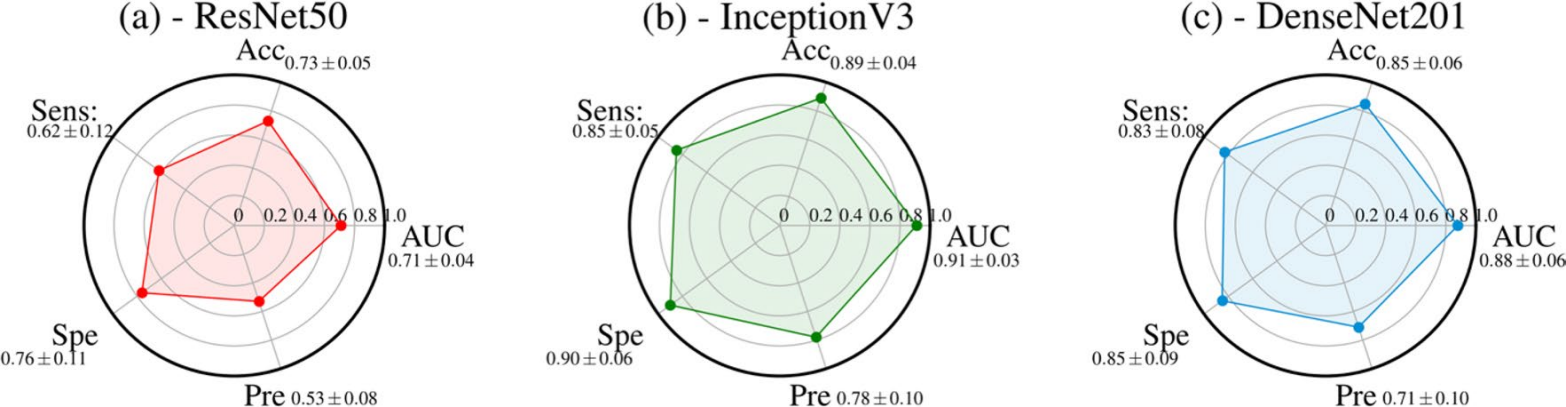
Radar plots of the performances AUC, Accuracy (Acc), Sensitivity (Sens), Specificity (Spe), and Precision (Pre) of three CNNs: ResNet50 (**a**), InceptionV3 (**b**), and DenseNet201 (**c**). For each metric, the mean value, among all the cross-validation 20 rounds, is shown with its standard deviation.

On the other hand, performances of CNNs are shown in the radar plots of Fig. 4. InceptionV3 (Fig. 4b) outperformed the other structures by achieving an AUC value, accuracy, sensitivity, specificity, and precision of 0.91 ± 0.03, 0.89 ± 0.04, 0.85 ± 0.05, 0.90 ± 0.06 and 0.78 ± 0.10, respectively.

**Figure 5:**
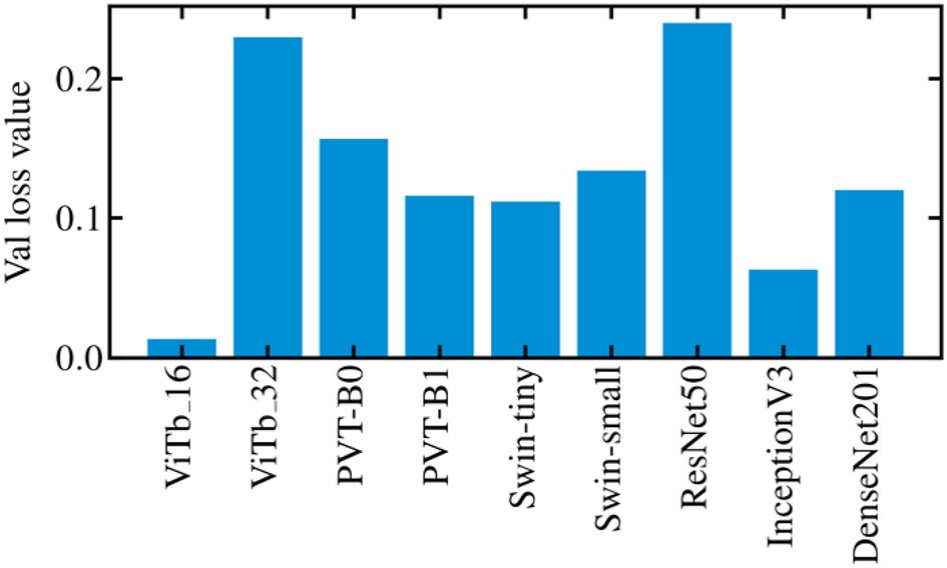
Example of the training loss function and validation loss plots as a function of the 30 epochs of training (**a**). Histogram of the validation loss values, averaged over all the epochs, rounds, and folds of the cross-validation for ViTs, PVTs, Swins, and CNNs (**b**).

As additional result, Fig. 5 shows a histogram of the validation loss values, averaged over all the epochs, folds and rounds of cross-validation, for ViTs, PVTs, Swins and CNNs. ViTb_16, PVT-B1, Swin-tiny, and InceptionV3 show the lowest validation loss in the histogram within their family. The best trade-off between the performances achieved and the loss valued was reached by InceptionV3.

## Discussion and conclusion

The a m of the study was to evaluate the performances of different deep learning algorithms for predicting recurrence in NSCLC patients by analyzing baseline CT. Our experimental results showed that ViTb_16, has higher performances, reaching an AUC and Accuracy values of 0.84 ± 0.04 and 0.83 ± 0.05, respectively, against ViTb_32, values equal to 0.67 ± 0.05 and 0.64 ± 0.08 respectively due to their different architectures. Indeed, ViTb_16 decomposes the input images into patches of size 16 × 16 pixels, while ViTb_32 into patches of size 32 × 32 pixels. Therefore, if the patch size is smaller, the transformer encoder’s attention would be higher, bringing to a better classification. As regards the Swin cases, both Swin-tiny and Swin-small are comparable (AUC = 0.82 ± 0.04 and 0.80 ± 0.07; Accuracy = 0.79 ± 0.04 and 0.77 ± 0.04 respectively). The best performances among the considered Transformers techniques are reached with PVT-B1 with the AUC and Accuracy value of 0.90 ± 0.04 and 0.86 ± 0.04 respectively. These better performances, among all considered Transformers, could depend on the PVT overlapping patch embedding mechanism allowing the Transformer to extract more information from the CT image than ViTs and Swins^29^. In the end, the best performances of this study are reached via pre-trained CNN InceptionV3 with AUC and Accuracy equal to 0.91 ± 0.03 and 0.89 ± 0.04 respectively. Even if CNNs perform best, the considered Transformers ViTs, PVTs and Swins still reach high and comparable performances.

As regards the topic of NSCLC classification, we scanned the literature and, to the best of our knowledge, we identified the state-of-the-art works which mainly use clinical features or radiomic ones. The latter can be further split into handcrafted features or extracted via CNNs^14, 49–52^. To the best of our knowledge, we use pre-trained ViTs, PVTs, and Swins for the first time, for the specific task of NSCLC classification. Table 1 summarizes the principal results proposed in the state-of-the-art according to the topic of our clinical task.

**Table 1:** Table of the state-of-the-art performances achieved in previous works about NSCLC recurrence prediction.

	N. of patients	Dataset	Model	Performances
Wang et al.^51^	157	Private	Handcrafted Radiomic features based	Acc = 0.85
Aonpong et al.^50^	88	Public	CNN + gene-expression based	AUC = 0.77 Acc = 0.83
Kim et al.^49^	326	Public	CNN based + Handcrafted Radiomic based + Clinical based	AUC = 0.77 Acc = 0.73
Hindocha et al.^52^	657	Private	Clinical based	AUC = 0.69
Bove et al.^14^	144	Public	CNN based + Clinical based	AUC = 0.83 Acc = 0.79
Our proposed model		Public	CNN + Transformer based	AUC = 0.91 Acc = 0.89
Our proposed model	144		ViT + Transformer based	AUC = 0.90 Acc = 0.86

S. Hindocha et al. predicted recurrence, recurrence-free survival, and overall survival of NSCLC patients, employing only clinical features from a cohort of 657 patients. As regards the task of recurrence prediction, an AUC equals to 0.69 was reached^51^. In the work of Wang et al., for example, CT images from a cohort of 157 NSCLC patients were analyzed using only handcrafted-radiomic features reaching an a ccuracy equals to 0.85^52^.

As regards NSCLC recurrence radiomic studies based on deep learning models, we mention the works of Aonpong et al., Kim et al., and Bove et al.^14, 49, 50^. In the former, Authors used a subsample of our same radiogenomic database to predict the NSCLC recurrence implementing a genotype-guided radiomic model focusing on a smaller cohort of 88 patients^50^. Using various state-of-the-art CNNs, gene expression data were extracted from CT images achieving an AUC equals to 0.77, and a ccuracy equals to 0.83. In the second one, Kim et al.^49^ built various ensemble-based prediction models using a database of 326 patients including our one. Clinical data, handcrafted radiomic features, and deep learning radiomic ones were considered and combined with each other. The best performances combining all together were AUC equals to 0.77, and Accuracy equals to 0.73. Finally, in the work of Bove et al. a transfer learning approach was implemented extracting radiomic features from the cropped CT images, around the tumor area, of our same NSCLC radiogenomic dataset^53^ via pre-trained CNNs, reducing the number of radiomic features and combining them with the clinical data of the database. The best reached performances consisted of AUC and Accuracy equal to 0.83 and 0.79 respectively^14^.

Considering all the results, in our model pre-trained CNN InceptionV3 seems to outperform the state-of-the-art works on NSCLC recurrence classification topic.

We would like to underline that the comparison with the state of the art is purely naïve. Unfortunately, the works proposed in the literature on the same clinical task have often been developed starting from private datasets. Even when they use the same public dataset to which we referred, the authors integrated the public data with private data (as in the work presented by Kim et al.^49^), without then differentiating the results obtained, or selected a subset of data according to certain criteria, which could be compatible with the objective of our work (as for the work presented by Aonpong et al.^50^). Therefore, it is difficult to make objective comparisons on the same dataset.

However, our model still suffers from some limitations. Indeed, although a data augmentation technique has been used to reinforce the training of the last layers of the pre-trained networks used, the obtained performances are strongly influenced by the retrospective nature and small dimension of the dataset. Specifically, the model needs to be validated in a more robust manner also using an external validation set, preferably referring to a sample of private data, although the use of a public database as is known allows an objective comparison of the proposed methods. Therefore, for the future, we intend to collect a larger database of NSCLC patients to validate and optimize the proposed models; moreover, we will also evaluate other public dataset to test the obtained results. Another possible future direction in the research would include a further investigation of more Transformer architectures and their correspondent performances. Moreover, further studies could include both combined deep radiomic and clinical features to train suitable Machine Learning classifiers to predict NSCLC recurrence after years with the help of the *Explainable Artificial Intelligence* (XAI) to detect the most relevant and decisive features for the prediction^54, 55^.

## Materials and methods

### Experimental dataset

In our work, we used a public radiogenomics dataset of NSCLC available in the Cancer Imaging Archive (TCIA)^53^. The public database consisted of 211 subjects divided into two sub-cohorts:

1. The R01 cohort with 162 patients (38 females and 124 males, age at scan: mean 68, range: 42–86) from Stanford University School of Medicine (69) and Palo Alto Veterans Affairs Healthcare System (93) recruited between April 7ths 2008 and September 15th, 2012;
2. The second AMC cohort consisting of 49 additional subjects (33 females, 16 males, age at scan: mean 67, range 24–80) was retrospectively collected from Stanford University School of Medicine based on the same criteria.

We chose to focus only on the (1) sub-cohort R01 because they had both tumor segmentation binary masks and the axial CT available. Among the 162 patients of cohort R01, the tumor segmentation mask was not available for 18 patients, so the final number of patients involved in this study is equal to 144, of which 40 (27.78%) with a recurrence event within eight years from the first diagnosis. For each patient, a CT image in DICOM format was available and was acquired by preoperative CT scans with a thickness of 0.625–3 mm and an X-ray tube current at 124–699 mA at 80–140 KVp. On the other hand, the related segmentations were defined on the axial CT image series by thoracic radiologists with more than five years of experience and adjusted using ePAD software^53^.

Beyond CTs and binary tumor masks, the adopted database includes the following clinical features: Recurrence (values: yes, no), age at histological diagnosis, weight, gender (values: female, male), histology (values: adenocarcinoma, squamous cell carcinoma, not otherwise specified), pathological T (values: T1, T2, T3, T4), pathological N stage (values: N0, N1, N2), histopathological grade (values: G1, G2 and G3), lymphovascular invasion (values: absent, present, not collected) and pleural invasion (values: yes, no)^53^. All these clinical features are listed in Table 2.

**Table 2:** Table of the clinical features of the adopted dataset and their distributions.

Clinical feature	Distribution
Recurrence	
Yes (abs; %)	(40; 27.78%)
No (abs; %)	(104; 72.22%)
Age at histological diagnosis	
Median [q_1_; q_3_]	69 [64; 76]
Weight (lbs)	
Median [q_1_; q_3_]	173.5 [145.13; 198.90]
Nan (abs; %)	(10; 6.94%)
Gender	
Female (abs; %)	(36; 25%)
Male (abs; %)	(108; 75%)
Histology	
Adenocarcinoma (abs; %)	(112; 77.77%)
Squamous cell carcinoma (abs; %)	(29; 20.14%)
Not otherwise specified (abs; %)	(3; 2.08%)
Pathological T stage	
T1 (abs; %)	(74; 51.39%)
T2 (abs; %)	(49; 34.03%)
T3 (abs; %)	(16; 11.11%)
T4 (abs; %)	(5; 3.47%)
Pathological N stage	
N0 (abs; %)	(115; 79.86%)
N1 (abs; %)	(12; 8.33%)
N2 (abs; %)	(17; 11.8%)
Histopathological grade	
G1 (abs; %)	(37; 25.69%)
G2 (abs; %)	(80; 55.56%)
G3 Poorly differentiated (abs; %)	(27; 18.75%)
Lymphovascular invasion	
Absent (abs; %)	(121; 84.03%)
Present (abs; %)	(18; 12.5%)
Not Collected (abs; %)	(5; 3.47%)
Pleural invasion	
No (abs; %)	(105; 72.92%)
Yes (abs; %)	(39; 27.08%)

“*Nan*” means “*Not A Number*” if the data is missing in the database, “*abs*” stands for “*absolute value*”.

In this study, the clinical data were not used, and the recurrence feature (yes = 1, no = 0) was chosen as a label for image classification.

**Figure 6:**
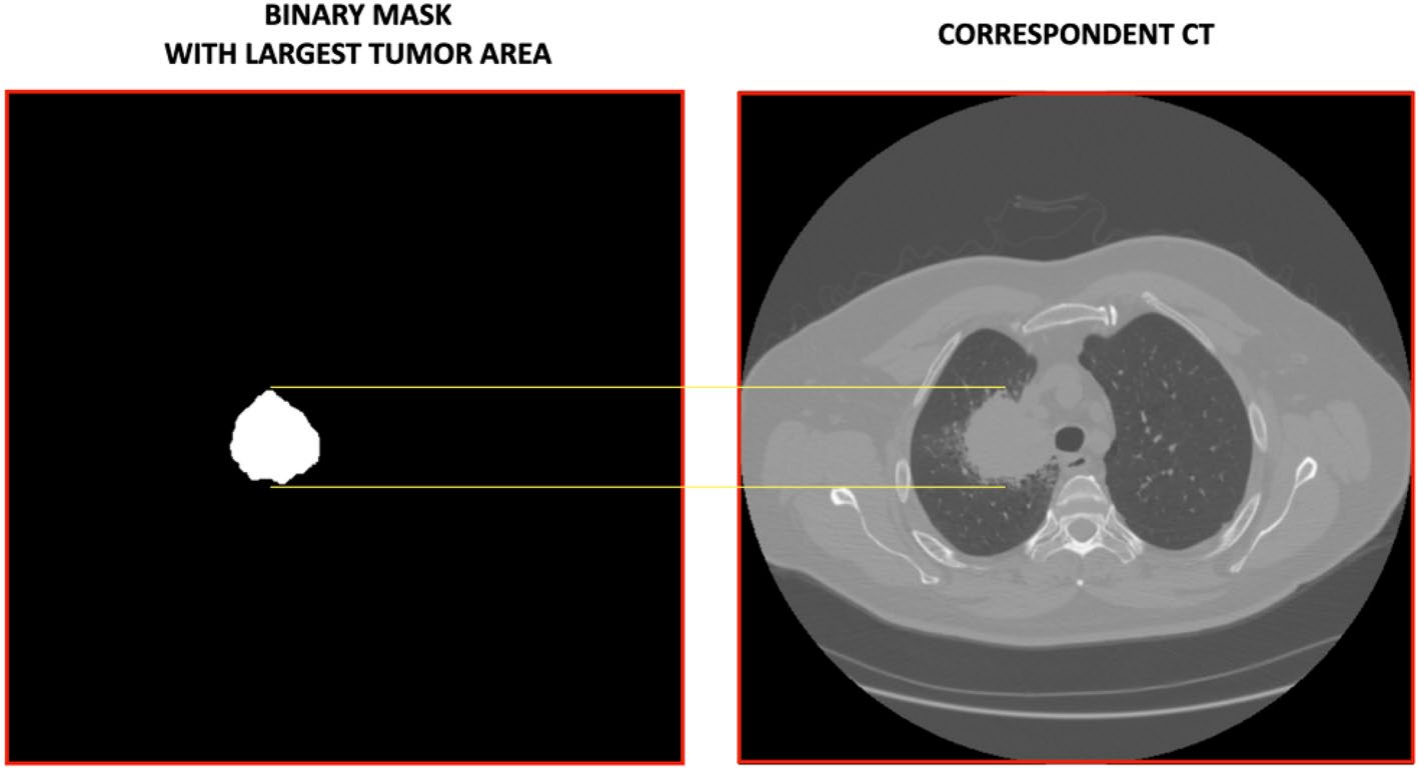
An example of a binary Mask with the largest tumor area and its corresponding CT are shown. The yellow lines mark the tumor area in the CT. For each patient, we detected this correspondence, and the CTs, suitably rescaled in the range [0;1] and with a specific input size, were then used as input for the ViTs, PVTs, Swins, and CNNs.

For each patient, we first detected the segmentation mask with the largest tumour area and found the corresponding CT slide for the analysis as shown in Fig. 6.

### ViTs, PVTs, Swins and CNNs architectures

After detecting the CTs with the largest tumor area, we adopted a deep learning transfer-learning approach involving pre-trained ViTs, PVTs, Swins, and CNNs. All the analysis steps were performed using Python programming language with Tensorflow-Keras^56, 57^.

First, the original CT image pixels were normalized in the range [0;1] and then reshaped to the specific input size of the Transformers and CNNs. Then, the whole pre-processed images became the input for the various models.

**Figure 7:**
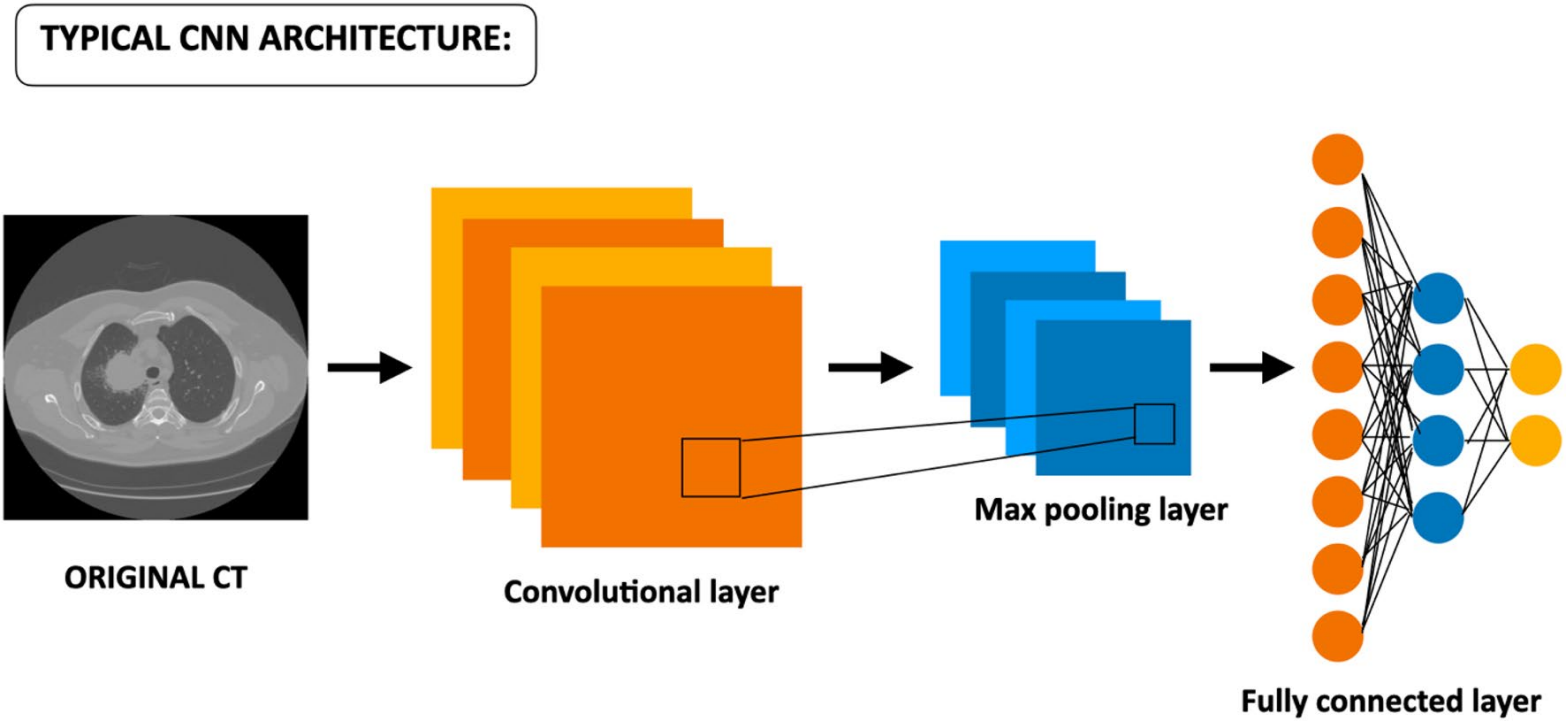
Typical architecture of a CNN. It takes the input image, suitably resized, and elaborates it through a series of internal layers consisting of convolutional ones, max-pooling layers, and fully connected layers until final classification^10–20^.

The usual architecture of state-of-the-art CNNs, shown in Fig. 7, consists of three key elements represented by the convolutional layers, the pooling layers, and the fully connected. Once the CNN receives an input image suitably pre-processed, the convolutional layers are the ones dedicated to learning features from the input images, instead, the max-pooling layers are responsible for the reduction of the size of feature maps. At the end of the CNN, fully connected layers are added in a stacked way which, via a specific function (e.g., SoftMax or Sigmoid), provides classification^10–20^.

**Figure 8:**
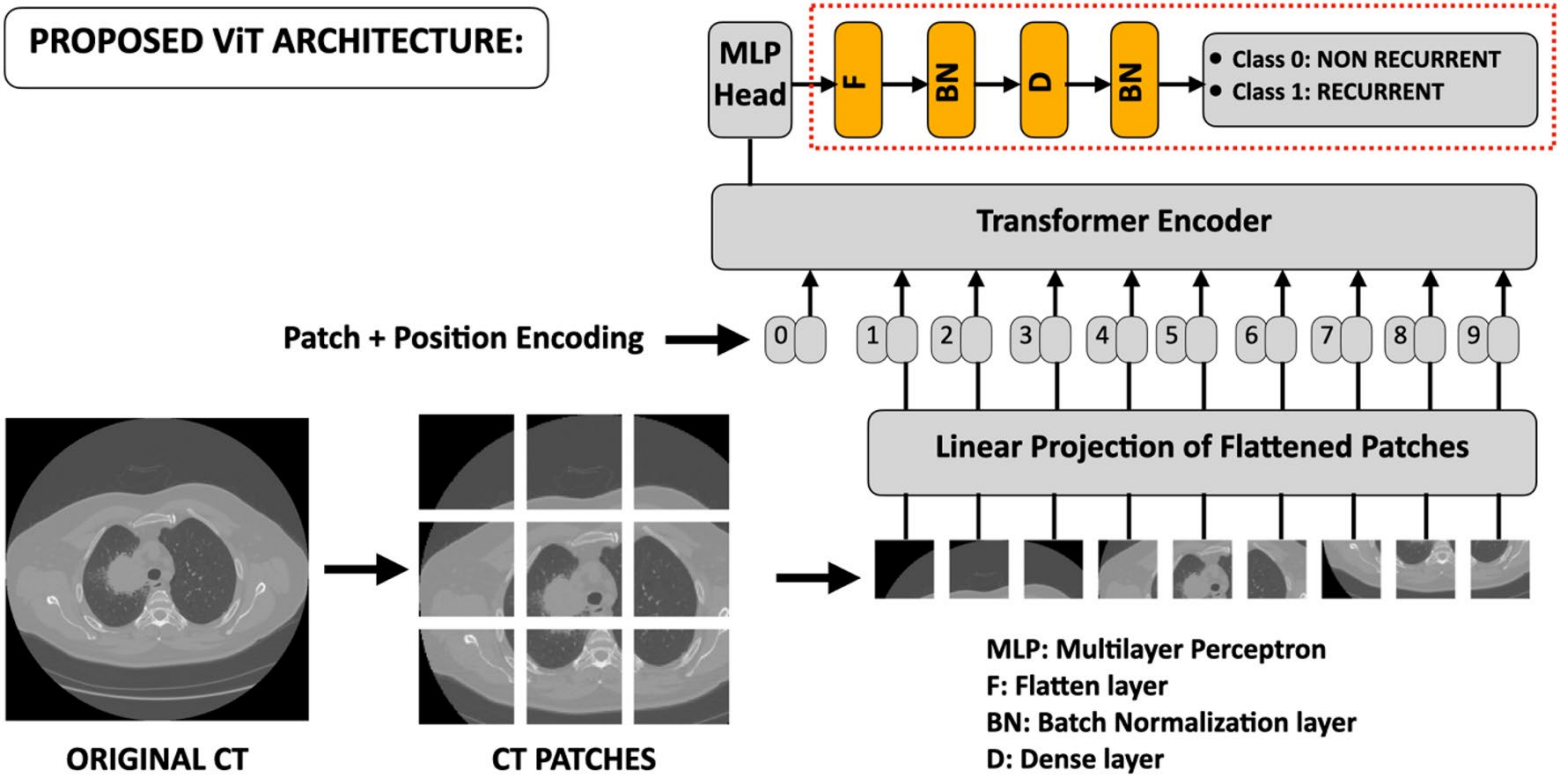
Proposed architecture of the ViTs starting from Dosovitskiy et al.^24^. The original input image, suitably pre-processed, is then decomposed into *N* patches then flattened obtaining a linear patch projection. Through the Transformer Encoder, these elements are sent to the head of MLP, which provides classification. The yellow final boxes placed after the MLP, inside the red dashed rectangle, indicate the new added layers of the proposed; these have also been adopted for the CNNs.

The architecture of the Transformers, shown in Fig. 8 according to the architecture of A. Dosovitskiy et al., is quite different from traditional CNNs^24^. ViTs derive from the original transformer model used in the natural language processing (NLP), where the input object consists of one-dimensional word tokens. The input images, of typical size 224 × 224 pixels, of height *H*, width *W*, and channels *C* are divided into smaller patches with number N = HW/P^2^ being *P* × *P* the pixel size of the input image. To perform the classification task, ViTs are equipped with an encoder that receives the sequence of embedded picture patches, together with positional data, and a learnable class embedding suspended sequence. The latter is sent to the classification head coupled to the output of the encoder. Therefore, the data sequence is the following:

- Original images are resized to size e.g., 224 × 224, and normalized between [0;1]. They are then decomposed in the* N* patches.
- The obtained patches are then flattened obtaining a linear patch projection.
- Learnable embeddings with patch projections are then concatenated. The positional embedding marks the order of the single patch in the sequence.
- The output of the transformer encoder is sent to a Multilayer perceptron head (MLP) that with additional layers of this work, e.g., a Flatten layer, a Batch Normalization layer, a Dense layer with 64 units, another Batch Normalization layer, and the final Dense layer with sigmoid function shown in red dashed box of Fig. 3, provide classification.

In this study, we performed different experiments using two ViT models: a base model with 16 × 16 image patch size (ViTb_16) and a base model with 32 × 32 image patch size (ViTb_32) both consisting of 12 hidden layers^22–24^. PVTs represent a variant of the original ViTs and as stated by their name, they possess a columnar pyramid structure similar to traditional CNNs^29, 30^. In this work, we adopted the improved version PVTs v2, from Wang et al. (2022), which introduced the linear complexity attention layer, the overlapping patch embedding and convolutional feed-forward network orthogonal to original PVTs. From now on, throughout the text, for the sake of simplicity, we will use the term PVT to indicate PVT v2 architecture of Wang et al.^29, 30^. We considered two models of this family: PVT-B0 and PVT-B1. Both consist of four stages characterized by C_i_ channel number of the output of stage i, R_i_ reduction ratio, N_i_ head number, E_i_ expansion ratio of the feed forward layer, and L_i_ number of encoder layers for i = 1–4 hyperparameters. For both L_1_–L_4_ equals 2 whereas C_i_, for i = 1–4, of PVT-B1 is double of the correspondent PVT-B0^29, 30^. The Swin Transformer is another Transformer architecture^27^. As the name states, *Shifted Window*, the key idea of this type of Transformer is to build a hierarchy starting from small-sized patches and gradually merging neighbouring patches into deep Tranformer layers. Between a self-attention layer and the next one, there is a window shift resulting in a new one. We adopted two types of this architecture consisting of the Swin-tiny and Swin-small which provided the best performances. The hyper-parameters of these types of Swins are represented by the channel number *C* of hidden layers in the first stage being C = 96 for both the Swin-tiny and small and the layer numbers being {2,2,6,2} ({2,2,18,2}) for the tiny one (small)^27^. For all the analyzed Transformers the ideal image size has been set to 224 × 224 pixels.

As regards traditional CNNs, we used three well-established state-of-the-art CNNs of different families: ResNet50, DenseNet201, and InceptionV3.

In Python Tensorflow-Keras, ResNet50 requires input images of size 224 × 224 pixels with 177 total layers. Differently, InceptionV3 needs input images of 299 × 299 pixels with 313 total layers. In the end, DenseNet201 accepts input images of 224 × 224 pixels size with a total of 709 layers^56, 57^.

### Learning model

We built transfer learning models using pre-trained ViTs, PVTs, Swins, and CNNs on the ImageNet natural image dataset to train the dataset of NCSLC patients to predict the recurrence event^56–58^. The application of transfer learning to ViT, PVT, and Swin architectures consisted in replacing the last layer with the following layers: a flattening layer plus a batch normalization, one dense layer with Gelu activation function followed by another batch normalization, and the final dense layer as classifier with a sigmoid activation function. The red dashed box in Fig. 8 shows the added layers. This scheme was also adopted for CNNs replacing the *Gelu* with the *Relu* activation function for the added dense layer. These new networks were then trained for the image classification task. We implemented a stratified tenfold cross-validation in 20 external rounds on the entire dataset of 144 patients. In each fold of the cross-validation, 90% of the dataset corresponding to 130 elements is used as a training set, whereas the remaining 10%, corresponding to 14 elements, is used as the test set.

In this study, all the models were trained for 30 epochs in each fold of the cross-validation with batch size equal to 10 elements. Adam optimizer with an initial learning rate of 10–4 was used to optimize the weights of the network. To handle the imbalancing of the dataset, a sigmoid focal cross-entropy was used as loss function with balancing factor α and modulating factor β equal 0.25 and 2.0 respectively^59^. Considering our database is relatively small, to make our analysis more robust we implemented a data augmentation process, in addition to the transfer-learning approach, using three built-in Keras transformations such as Random Flip, Random Rotation, and Random Contrast^57^. This data augmentation was added as an additional layer in the models.

After the training phase, the model was used to predict the probability scores and then used to compute the performances via the Scikit-learn library functions^58^. Performances of classification of NSCLC recurrence for pre-trained ViTs, PVTs, Swins and CNNs have been evaluated in terms of the Area Under the Curve (AUC), Accuracy, Sensitivity, Specificity, and Precision. These metrics are computed in each of the 20 rounds of the stratified cross-validation so, in the end, the final performances, of the specific model, are evaluated as an average of all the 20 values with their corresponding standard deviation. To better balance these metrics, a Youden index test was performed^60^.

## Data Availability

The data was obtained from the open-access NSCLC-Radiogenomics dataset publicly available at The Cancer Imaging Archive (TCIA) database (https://wiki.cancerimagingarchive.net/display/Public/NSCLC+Radiogenomics). Imaging and the clinical data have been de-identified by TCIA and approved by the Institutional Review Board of the TCIA hosting institution. Ethical approval was reviewed and approved by Washington University Institutional Review Board protocols. Informed consent was obtained from all individual participants included in this study^53^. The source codes can be found at the following link: https://github.com/mcomes92/NSCLC_Vit_CNN.
